# Calnexin cycle – structural features of the ER chaperone system

**DOI:** 10.1111/febs.15330

**Published:** 2020-04-27

**Authors:** Guennadi Kozlov, Kalle Gehring

**Affiliations:** ^1^ From the Department of Biochemistry & Centre for Structural Biology McGill University Montréal QC Canada

**Keywords:** calnexin, calnexin cycle, calreticulin, CypB, endoplasmic reticulum, ERp29, ERp57, PDI, protein folding, UGGT

## Abstract

The endoplasmic reticulum (ER) is the major folding compartment for secreted and membrane proteins and is the site of a specific chaperone system, the calnexin cycle, for folding N‐glycosylated proteins. Recent structures of components of the calnexin cycle have deepened our understanding of quality control mechanisms and protein folding pathways in the ER. In the calnexin cycle, proteins carrying monoglucosylated glycans bind to the lectin chaperones calnexin and calreticulin, which recruit a variety of function‐specific chaperones to mediate protein disulfide formation, proline isomerization, and general protein folding. Upon trimming by glucosidase II, the glycan without an inner glucose residue is no longer able to bind to the lectin chaperones. For proteins that have not yet folded properly, the enzyme UDP‐glucose:glycoprotein glucosyltransferase (UGGT) acts as a checkpoint by adding a glucose back to the N‐glycan. This allows the misfolded proteins to re‐associate with calnexin and calreticulin for additional rounds of chaperone‐mediated refolding and prevents them from exiting the ERs. Here, we review progress in structural studies of the calnexin cycle, which reveal common features of how lectin chaperones recruit function‐specific chaperones and how UGGT recognizes misfolded proteins.

## Graphical abstract

The endoplasmic reticulum is the major folding compartment for secreted and membrane proteins and is the site of a specific chaperone system, the calnexin cycle, for folding N‐glycosylated proteins. Here, we review progress in structural studies of the calnexin cycle, which reveal common features of how lectin chaperones recruit function‐specific chaperones and how UGGT recognizes misfolded proteins.

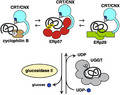

**Abbreviations**
CNX: calnexin
CRT: calreticulin
UGGT: UDP‐glucose:glycoprotein glucosyltransferase
CypB: cyclophilin B
PDI: protein disulfide isomerase
ER: endoplasmic reticulum

## Introduction

The endoplasmic reticulum (ER) contains two major folding pathways for protein substrates [1]. One is the general folding pathway, and one is specific for glycoproteins. The general pathway is mostly mediated by BiP, the ER homolog of 70‐kDa heat shock protein (Hsp70), and P4HB (PDIA1), the founding member of the protein disulfide isomerase (PDI) family. BiP acts as a general chaperone, while P4HB and other PDIs promote the formation of protein disulfides through the action of thioredoxin‐like domains that catalyze oxidation and isomerization of disulfides [2, 3, 4].

**Fig. 1:**
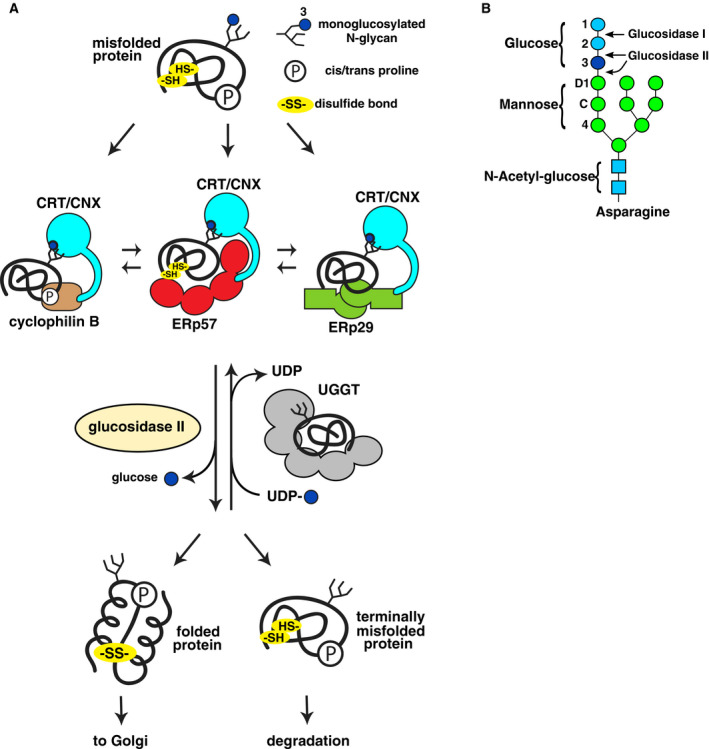
Overview of calnexin/calreticulin cycle. (A) The monoglucosylated form of newly synthesized glycoproteins proteins binds to calreticulin (CRT)/calnexin (CNX) and promotes protein folding with assistance from ERp57, CypB, and ERp29. Following release of the terminal glucose by glucosidase II, natively folded proteins are transported to Golgi. Incompletely folded proteins are reglucosylated by UGGT and rebind calreticulin/calnexin for additional folding cycles. If multiple folding cycles are unsuccessful, terminally misfolded proteins are transported to the cytoplasm for degradation via the ER‐associated protein degradation (ERAD) pathway. (B) Structure of N‐linked glycan. The precursor glycan is attached to the protein with three glucose residues. The first two are removed through the action of glucosidases I and II to generate the monoglucosylated form that is required for binding calnexin and calreticulin. UGGT acts on misfolded glycoproteins to add back glucose to the glycan for additional rounds of chaperone‐mediated folding.

The pathway dedicated for N‐glycosylated proteins is named after calnexin, the first protein discovered in the pathway [5]. Upon entering the ER, N‐linked glycoproteins have specific asparagines labeled with a Glc_3_Man_9_GlcNAc_2_ glycan. Calnexin (also called IP90, major histocompatibility complex class I antigen‐binding protein p88, or p90) is one of four lectin chaperones in the ER. Calnexin and its soluble homolog, calreticulin, combine a lectin‐like glycan‐binding domain with a flexible arm, the P‐domain that recruits other chaperones. The other major components of the pathway are UDP‐glucose:glycoprotein glucosyltransferase (UGGT), the protein disulfide isomerase ERp57, and the ER glucosidases Glu I and Glu II [6, 7, 8] (Fig. 1A).

Protein folding in the calnexin cycles starts with protein synthesis and N‐glycosylation as the protein enters the ER. The N‐glycan is then trimmed by glucosidase I and glucosidase II to remove the outer and middle glucose residues, respectively, and generate the monoglucosylated form that specifically binds to calnexin or calreticulin [9, 10] (Fig. 1B). Through their P‐domains, the lectin chaperones bind function‐specific chaperones [11, 12, 13, 14, 15, 16], which act on the bound glycoprotein to promote its folding and maturation. Glucosidase II is capable of removing the remaining glucose moiety. When this occurs, the glycoprotein is no longer able to bind calnexin/calreticulin, ending the first round of glycoprotein folding. If protein has not yet adopted its native conformation, the glucosyltransferase UGGT adds back the last glucose residue to allow the glycoprotein to bind again to calnexin/calreticulin. In this way, UGGT acts as a quality control system by specifically recognizing misfolded Man_9_GlcNAc_2_ glycoproteins and returning them to calnexin/calreticulin for further processing.

Three different function‐specific ER chaperones are known to bind to calnexin/calreticulin. ERp57 is a protein disulfide isomerase and catalyzes the oxidation and isomerization of glycoprotein disulfide bonds. The two other chaperones, cyclophilin B (CypB) and ERp29, carry out the isomerization of peptide bonds and a general chaperone function, respectively.

There are intricate relationships between calnexin cycle and antigen presentation pathways. Calnexin cycle proteins calreticulin and ERp57 do not only chaperone MHC class I heavy chains, but are also a part of the peptide‐loading complex (PLC), which also includes transporter associated with antigen processing (TAP), β2‐microglobulin, and tapasin. PLC is required for loading of antigenic peptides onto MHC class I heavy chains for presentation to the immune system [17]. UGGT plays a role in this pathway by surveying the loading of MHC class I complexes, including reglucosylation of empty complexes [18, 19]. It also reglucosylates incorrectly assembled T‐cell antigen receptor (TCR) complexes [20].

This review provides an overview of proteins involved in calnexin cycle with an emphasis on recent structural insights (Table 1). These include calreticulin in the context of PLC, P‐domain recognition by ER chaperones, and structural characterization of UGGT [15, 21, 22, 23, 24, 25]. As recent structures of glucosidase II were well covered in other reviews [26, 27], this work will focus on developments in understanding of UGGT and calreticulin and their mechanism of action.

**Table 1:** Structures of calnexin cycle proteins.

Protein/complex	Species	PDB code	Resolution (Å)	Comments
Lectin chaperones				
Calnexin	*Canis lupus familiaris*	1JHN	2.9	Luminal domain [28]
Calreticulin	*Mus musculus*	3RG0	2.57	Lectin domain with a partially truncated P‐domain [82]
Calreticulin	*M. musculus*	3O0W	1.95	Lectin domain in complex with Man_3_Glc_1_ tetrasaccharide [29]
Calreticulin	*M. musculus*	3O0X 3O0V	2.0 2.3	Lectin domain [29]
Calreticulin	*Homo sapiens*	3POW 3POS	1.55 1.65	Lectin domain [41]
Calreticulin	*Rattus norvegicus*	1HHN	n/a	NMR structure of the P‐domain [47]
Calreticulin	*R. norvegicus*	1K91	n/a	NMR structure of truncated P‐domain [116]
Calreticulin	*Entamoeba histolytica*	5HCA 5HCB	2.15 2.9	Lectin domain in complex with glucose [117]
Calreticulin	*Trypanosoma cruzi*	5HCF	2.45	Lectin domain [117]
MHC class I peptide loading				
Peptide‐loading complex	*H. sapiens*	6ENY	5.8	Cryo‐EM structure of PLC‐editing module [21]
Tapasin/ERp57	*H. sapiens*	3F8U	2.6	Full‐length proteins [56]
P‐domain complexes				
CypB	*H. sapiens*	3ICI	1.7	Complex with calmegin P‐domain [14]
ERp29	*H. sapiens*	5V8Z	2.1	ERp29 C‐term with calmegin P‐domain [15]
ERp29	*H. sapiens*	5V90	3.2	ERp29 C‐term with calreticulin P‐domain [15]
Glucosyltransferases				
Full‐length UGGT	*Chaetomium thermophilum*	5MZO	3.48	Open conformation [22]
Full‐length UGGT	*Ch. thermophilum*	5N2J	4.4	Closed conformation [22]
Full‐length UGGT	*Ch. thermophilum*	5MU1 6TRF	3.48 4.11	Intermediate conformation [22, 25]
UGGT folding sensor region	*Thermomyces dupontii*	5Y7O	3.1	TRXL and β‐sandwich domains [24]
UGGT catalytic domain	*Th. dupontii*	5H18	1.4	Complex with UDP‐glucose [24]
UGGT catalytic domain	*Ch. thermophilum*	6FSN	1.19	Complex with UDP‐glucose

## Structure of calnexin/calreticulin

**Fig. 2:**
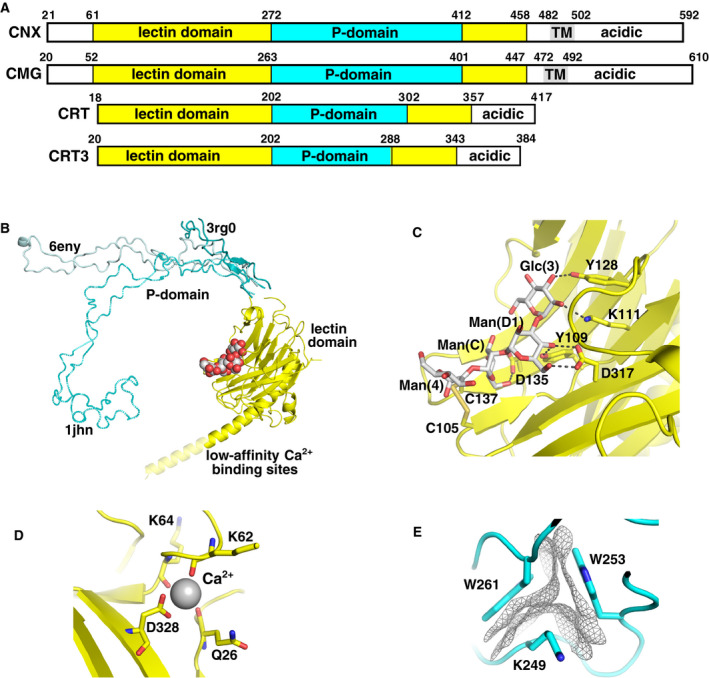
Calnexin/calreticulin structure. (A) Domain architecture of calnexin (CNX), calmegin (CMG), calreticulin (CRT), and calreticulin 3 (CRT3). The P‐domain is inserted into the lectin domain, while calnexin and calmegin also possess a transmembrane (TM) domain. The P‐domain in calnexin and calmegin is composed of four repeated modules, while the domain in calreticulin and calreticulin 3 contains only three modules. (B) Overlay of calnexin (PDB 1JHN) and calreticulin (PDB 3RG0 and 6ENY) structures illustrates flexibility of the P‐domain and the site of the glycan (red/white) bound to the lectin domain. In the peptide‐loading complex (PDB 6ENY), the C terminus of calreticulin forms a long helix, but it is unlikely to be folded in solution. The C termini of both proteins are rich in acidic residues that bind Ca^2+^ ions. (C) Four sugars of the glycoprotein glycan bind to the lectin domain along the β‐sheet surface (PDB 3O0W). The sugar‐binding specificity arises from the numerous hydrogen bonds between the glycan and protein. A disulfide bridge between Cys105 and Cys137 interacts with the Man(4) moiety. (D) High‐affinity Ca^2+^‐binding site in the lectin domain of calreticulin (PDB 3O0W). (E) Each repeated module in the P‐domain contains a small hydrophobic core of two tryptophans and a lysine residue. Residues from the calreticulin P‐domain structure (PDB 5V90) show the close packing of the hydrophobic van der Waals surfaces.

Calnexin and calreticulin are the most abundant representatives of a small family of lectin chaperones residing in the ER. The other members are the tissue‐specific homologs calmegin and calreticulin 3. Proteins in this family share common structure consisting of a glycan‐binding lectin domain and a very unusual arm‐like structure, termed the P‐domain due to the abundance of proline residues (Fig. 2A). The lectin domain adopts a globular fold with the P‐domain inserted in the middle of the lectin domain primary sequence [28]. Two of the lectin chaperones, calnexin and calmegin, are membrane‐bound through a C‐terminal transmembrane helix, while the calreticulins are soluble proteins. Calnexin has a C‐terminal cytosolic RKPPRRE motif involved in the endoplasmic reticulum retention, while calreticulin possesses a luminal KDEL‐retrieval sequence.

Structures of the lectin domains of calnexin and calreticulin (Table 1) show a jellyroll fold largely formed by a sandwich of two large β‐sheets: a seven‐stranded, concave β‐sheet and a six‐stranded, convex β‐sheet. Besides additional small β‐sheet and two short α‐helices (Ala32–Arg36 and Leu196–Asp199), a prominent feature of calreticulin is a long C‐terminal α‐helix (Glu336–Asp362) that runs along and beyond the convex β‐sheet (Fig. 2B). The recent cryo‐EM structure of the peptide‐loading complex containing full‐length calreticulin modeled this helix extending until Glu386 with ~ 30 missing residues due to disorder [21]. It appears likely that the crystal structures provide a more realistic view of folded boundaries in solution, as ~ 20 C‐terminal residues in the cryo‐EM structure are modeled without sufficient electron density. In agreement with that, limited proteolysis experiment readily yielded cleavage at Lys368, suggesting that the folded region ends prior to that residue [29]. It should be noted that while the C‐terminal tail is unlikely to produce a stable structure in solution, it might become more ordered upon binding calcium ions [30].

The details of glycan binding were revealed by the high‐resolution structure of calreticulin in complex with Glc_1_Man_3_ tetrasaccharide, the Glc(3)‐Man(D1)‐Man(C)‐Man(4) branch of the monoglucosylated Glc_1_Man_9_GlcNAc_2_ glycan [29] (Fig. 2C). The tetrasaccharide binds along the long groove formed by the curved β‐sheet with all sugar moieties engaged in protein binding. Importantly, the glucose moiety lies flat in the shallow cavity, the base of which is formed by Met131 and Ile147. In addition to these hydrophobic contacts, every oxygen of the glucose Glc(3) is involved in direct or indirect hydrogen bonds with the lectin domain, thus providing the specificity for glucose. The most crucial hydrogen bond is between O2 of Glc(3) and the side chain of Lys111 [29]. Mutagenesis studies have shown that Lys111 is required for the calreticulin–carbohydrate interaction [31, 32].

Man(D1) and Man(C) mainly use their O4–O6 edges for interactions with the lectin domain. In particular, O4 of Man(D1) engages in three direct hydrogen bonds with Tyr109 and both the side chain and backbone carbonyl of Asp317. Asp317 is required for binding because it also makes direct hydrogen bonds with O4 and O6 of Man(C) (Fig. 2C). The affinity of Glc_1_Man_3_ to the calreticulin lectin domain is 0.7 µm, which is very close to the reported value for intact calreticulin [33] suggesting that glycan binding is major route for substrate recognition by lectin chaperones.

The glycan‐binding surface is essentially identical in calnexin and calreticulin. The residues that are involved in carbohydrate binding are highly conserved and adopt very similar conformations in both proteins. In the cell, calnexin and calreticulin display overlapping but distinct patterns of interaction with substrate glycoproteins [34, 35, 36]. Because the calnexin/calreticulin lectin sites are nearly identical, the observed differences in substrate specificity must be based on other properties. Previous studies have shown that the distinct luminal versus membrane‐bound topologies of calreticulin and calnexin affect selection of substrate glycoproteins [34, 37, 38].

The lectin domains of both calreticulin and calnexin contain a solvent‐exposed disulfide bridge on the edge of lectin site. Previous studies showed that treatment with reducing agents dithiothreitol and tris(2‐carboxyethyl)phosphine (TCEP) abrogates carbohydrate binding by calreticulin [29, 39]. These cysteines are also essential to the chaperone function of calreticulin [40]. This is because this disulfide bond is involved in contacting the Man(C) and Man(4) moieties of glycan (Fig. 2C) [29].

The calreticulin lectin domain structures also defined the location of a high‐affinity calcium‐binding site [29, 41]. The calcium ion is coordinated by the side chain of Asp328, and backbone carbonyls of Gln26, Lys62, and Lys64 (Fig. 2D). Besides the high‐affinity site, the C‐terminal tail of calreticulin contains multiple low‐affinity Ca^2+^‐binding sites [42] and is responsible for high‐capacity Ca^2+^ storage in the ER [43]. Likewise, the highly acidic N‐terminal and C‐terminal regions of calnexin also contain multiple low‐affinity calcium‐binding sites [44]. More recent studies demonstrated that the C terminus of calreticulin has a propensity to form a helical structure [45] and its secondary structure gets enhanced in the presence of Ca^2+^ ions [45, 46].

## P‐domains

Sequence identify among the lectin chaperones is highest in the P‐domains. The domains are hairpin‐like structures composed of multiple type I and type II motif repeats [28, 47]. The calnexin and calmegin P‐domains are ~ 140 residues long and composed of four type I motifs IxDPxxxKP(E/D)DWD followed by four type II motifs GxWxxxxIxNP. The domains from calreticulin and calreticulin‐3 are smaller with only three repeats of each motif. The reason for that difference is unclear. It could reflect specificity for different protein substrates, or it could be due to fitting requirements into calnexin‐ and calreticulin‐specific multiprotein complexes. While calreticulin is best known for its involvement in MHC class I assembly and calnexin/calreticulin cycle in the endoplasmic reticulum, a multitude of recent studies demonstrated calreticulin expression on cell surface, where it appears to play a role in apoptosis and phagocytosis of dying cells (for a review, see Raghavan et al. [48]).

In the folded P‐domain structure, the type I motifs interact with type II in a head‐to‐tail fashion forming four modules each containing a small hydrophobic core of two tryptophans and a lysine (Fig. 2E). The hairpin‐like structure is additionally stabilized via interactions of conserved isoleucines producing an isoleucine zipper. In addition to being shorter, the calreticulin P‐domain is missing a disulfide bond (Cys360–Cys366) in the beginning of the tip module of calnexin and calmegin. The importance of this disulfide is unknown, but its reduction leads to local unfolding in the calnexin P‐domain (G. Kozlov, unpublished observations).

## Complexes of calnexin/calreticulin with ER chaperones

### ERp57

Cooperative interactions of chaperones are crucial for efficient protein folding in the ER. Calnexin and calreticulin often serve as a scaffold bringing together N‐glycosylated proteins with the ER‐resident chaperones. ERp57, a protein disulfide isomerase, was one of these proteins originally identified and established as a part of calnexin cycle pathway [11, 49]. More recent studies revealed and characterized interactions of lectin chaperones with cyclophilin B, a peptidyl‐prolyl isomerase, and a general chaperone ERp29 [14, 15].

ERp57 (also called ER‐60, GRP58, and PDIA3) possesses oxidoreductase activity [50, 51, 52] but becomes most active in combination with calnexin/calreticulin [11]. The physical association between ERp57 and calnexin/calreticulin has been demonstrated by cross‐linking [49, 53] and NMR [12, 13].

**Fig. 3:**
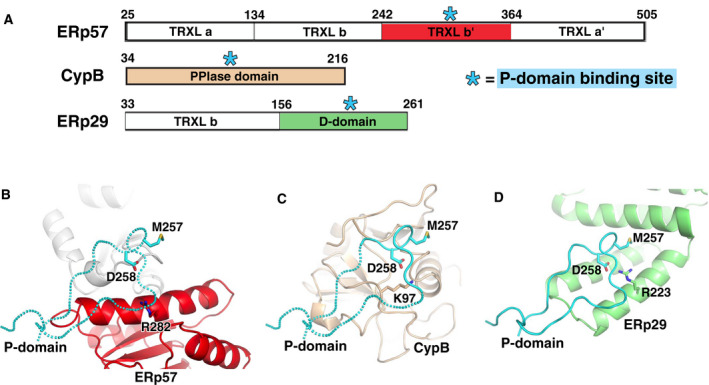
Interactions of calnexin/calreticulin with other ER proteins. (A) Domain architecture of calnexin/calreticulin‐binding partners. The asterisks mark domains required for interactions with calnexin/calreticulin. The tip of P‐domain (cyan) interacts with very different structural scaffolds from protein disulfide isomerase ERp57 (B), *cis*‐*trans* prolyl isomerase CypB (C), and general chaperone ERp29 (D). Met257 and Asp258 are part of the conserved Met‐Asp‐Gly sequence at the tip of P‐domains instrumental for the binding. Positively charged residues Arg282 (ERp57), Lys97 (CypB), and Arg223 (ERp29) indispensable for interactions with calnexin/calreticulin are also shown as sticks. Crystal structure of calreticulin P‐domain (PDB 5V90) was overlaid with cryo‐EM structure of P‐domain bound to ERp57 (PDB 6ENY) and with a shorter segment of P‐domain bound to CypB (PDB 3ICI). The modeled parts of P‐domains are shown with dashed lines. The ERp57‐P‐domain model was assembled using the high‐resolution structure of ERp57 **bb′** domain fragment (PDB 2H8L) and relative orientation of both proteins in cryo‐EM structure.

Structurally, ERp57 consists of four thioredoxin‐like (TRXL) domains termed **a**, **b**, **b′**, and **a′**. The N‐ and C‐terminal **a** and **a′** domains contain CGHC catalytic motifs, while the **b** and **b'** domains have lost the catalytic cysteines (Fig. 3A). ERp57 is similar to PDI both in its domain organization and the primary sequence. Similarity is highest in the catalytic **a** and **a′** domains (~ 50% identity) and lowest in the **b** and **b′** domains (~ 20%).

**Fig. 4:**
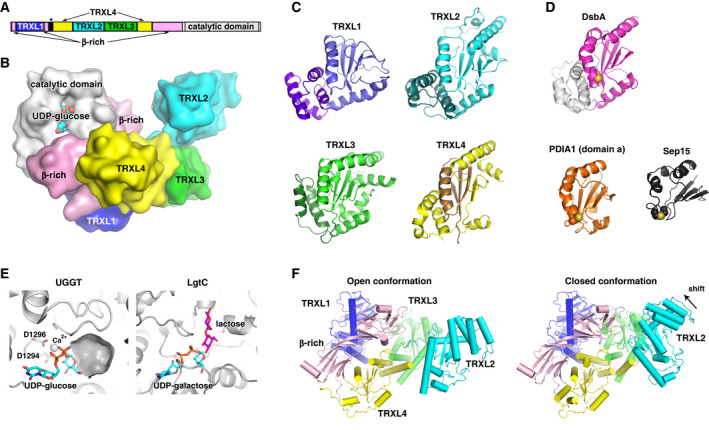
Structure of UDP‐glucose:glycoprotein glucosyltransferase. (A) Domain architecture of UGGT. The Sep15‐binding site is marked with an asterisk. (B) Cartoon representation of UGGT full‐length structure from *Chaetomium thermophilum* (PDB 5MZO) shows four DsbA‐like domains TRXL1 (blue), TRXL2 (cyan), TRXL3 (green), and TRXL4 (yellow) followed by β‐rich domain (pink) and catalytic domain (gray). Location of UDP‐glucose is modeled from the structure of the *Thermomyces dupontii* UGGT catalytic domain with bound UDP‐glucose (PDB 5H18). DsbA‐like domains of UGGT (C) have stronger resemblance to bacterial oxidoreductase DsbA (PDB 5KBC; magenta/gray) (D) than to thioredoxin‐like domains in mammalian PDI (PDB 3F8U; orange). Surprisingly, the helical bundle (purple) is at the N terminus of TRXL1 domain, while the TRXL4 domain is assembled from nonconsecutive stretches (yellow and brown) of the primary sequence. The C‐terminal domain of Sep15 (PDB 2A4H; dark gray) represents a minimalist version of a redox fold. (E) The UGGT catalytic domain is similar to other glycosyltransferases**.** The structure of UGGT catalytic domain (PDB 5H18) with bound UDP‐glucose (cyan) suggests that the acceptor glycan in UGGT binds in a surface cavity formed by a distorted helix next to the glucose moiety of UDP‐glucose. In the structure of the GT8 glycosyltransferases LgtC (PDB 1GA8), this pocket is occupied by the acceptor lactose (purple). Two aspartates in the UGGT catalytic site coordinate a calcium ion. (F) Interdomain mobility of UGGT. Comparison of the open (PDB 5MZO) and closed (PDB 5N2J) UGGT structures of *Ch. thermophilum* UGGT shows significant differences in positions of the TRXL2 and TRXL3 domains. The catalytic domain has been omitted to better show the conformational changes.

The thioredoxin‐like fold **TRXL = βαβαβαββα **(Pfam Thioredoxin_6 family PF13848) is a very stable and common domain consisting of a central five‐stranded β‐sheet covered by two α‐helices on each side. It is a derivative of classical thioredoxin fold **TRX = βαβαββα** (Pfam Thioredoxin family PF00085). Approximately twenty proteins in the large family of protein disulfide isomerases contain at least one thioredoxin‐like domain with a βαβαβαββα sequence of secondary elements. Some are catalytically (redox) active domains containing CxxC motif at the N terminus of helix 2, while noncatalytic domains do not contain catalytic cysteines and either play structural role or are involved in protein interactions. The bacterial thiol disulfide oxidoreductase, DsbA, that functions analogously to PDIs, displays a modified version of thioredoxin fold, a DsbA‐like thioredoxin fold **DSBA = βαβ–αααα–αββα** (Pfam DSBA family PF01323) with an extra four‐helical subdomain that caps one side of the domain (Fig. 4).

The extensive presence and diverse functions of thioredoxin‐like domains in the ER are quite remarkable (Table 2). What is the reason for the presence of this fold in so many proteins? One reason is that these domains are very robust and able to withstand a wide range of changes in environment. Another reason could be a versatility of this fold. Even within one protein (for instance PDI itself), the same fold can be utilized for oxidoreductase activity and for substrate binding.

**Table 2:** Thioredoxin‐like, DsbA‐like, and redox domains in the ER.

Protein family	Members	Function of the domains
Oxidoreductases	PDI, PDIp, ERp57, ERp72, ERp44, ERp46, PDIR, P5, ERdj5, ERp19, AGR2, AGR3	Thiol oxidation/reduction, protein binding
Transmembrane oxidoreductases	TMX, TMX3, TMX4, TMX5	Thiol oxidation/reduction
Chaperones	ERp29, PDILT, ERp27	Dimerization, protein binding
Glucosyltransferases	UGGT1, UGGT2	Structural role, protein binding
Selenoproteins	Sep15, SelM, SelT	Thiol reduction

Previous studies using NMR spectroscopy and mutagenesis revealed that the tip of the P‐domain of calnexin/calreticulin binds to ERp57 [12, 13, 54], while a large positively charged patch of residues in the ERp57 **b′** domain represents the calnexin/calreticulin‐binding site [55]. In particular, mutating Asp347 and Met346 of human calnexin (Asp258 and Met257 of calreticulin) completely abrogates the binding, as does the R282A mutation in ERp57. Furthermore, the K214A, K274A, and R282A mutants of full‐length ERp57 are compromised in their ability to fold monoglucosylated RNase B in *in vitro* folding assay, demonstrating a requirement for the calnexin–ERp57 interaction for efficient glycoprotein folding [55].

A visualization of calnexin/calreticulin–ERp57 interaction eluded numerous co‐crystallization attempts for a long time. Recently, a low‐resolution snapshot of the calreticulin–ERp57 complex in the context of peptide‐loading complex was obtained by cryo‐EM [21]. In that structure, the tip module of the P‐domain primarily interacts with the **b′** domain of ERp57 (Fig. 3B). The binding site comprises the N‐terminal half of long helix α2, the region preceding helix α4 of the **b′** domain, and the unusually long β4–β5 loop of the **b** domain. As a result of the interaction, the catalytic sites of ERp57 are facing the glycan‐binding site of calreticulin.

It is important to note that the structure represents only one of the possible orientations between calreticulin and ERp57 because of the intrinsic mobility of the P‐domain. The relative orientation of calreticulin and ERp57 in the peptide‐loading complex is mostly constrained by tapasin, which plays a role of pseudosubstrate of ERp57 by engaging its catalytic sites, while also interacting with the C‐terminal helix of calreticulin. The ERp57–tapasin positioning in the cryo‐EM structure is similar to the previously determined crystal structure of these proteins [56]. In the context of protein folding, the P‐domain flexibility would result in widening or narrowing the distance between the catalytic sites of ERp57 and the lectin site of calreticulin/calnexin. One of the implications would be an ability to adjust to protein substrates of variable sizes. On the other hand, this movement could be a driving force for unfolding the bound substrate, a necessary step in disulfide reshuffling.

It should be noted that the precise ERp57:CRT‐binding determinants are still to be resolved. While the structure confirms the binding sites on both proteins, its low resolution (5.8 Å) precludes us from identifying individual contacts responsible for the interaction. Moreover, the exact placement of the P‐domain relatively to ERp57 needs to be adjusted. This conclusion follows from steric clashes for a number of residues upon restoring their side chains missing in the model, for instance Lys274 of ERp57. Secondly, the structure does not explain the role of critical residues (such as Met257 and Asp258 of calreticulin among others), which are required for the binding. Therefore, a high‐resolution structure of the complex would be very informative in pinpointing structural determinants of the binding.

### CypB

Cyclophilin B (CypB) is a peptidyl‐prolyl cis‐trans isomerase (PPIase) found in the ER [57, 58] and inhibited by cyclosporin A binding to its active site with high affinity [57] (Fig. 3A). The functional relevance of cyclophilin B in the ER is demonstrated by its involvement in the folding of collagen [59] and the maturation of transferrin [60]. CypB expression is activated by the ER stress, whereas its absence makes cells more sensitive to ER stress [61].

The crystal structure of CypB in complex with the P‐domain from calmegin provided a mechanism for recruitment of PPIase activity to misfolded N‐glycoproteins and suggested that CypB functions as part of the calnexin cycle [14]. The structure shows that the tip of the P‐domain binds to a well‐defined surface opposite the cyclosporin A‐binding site and with a pronounced positively charged character due to the presence of multiple lysine residues (Fig. 3C). Comparison of structures of CypB in complex with cyclosporin A and the P‐domain shows that the prolyl isomerization and the calnexin/calreticulin‐binding sites are independent. Also, binding of the P‐domain is not affected by cyclosporine A bound to the active site of CypB, confirming that these binding events are functionally independent [14].

The single most important residues from each protein are Lys97 of CypB and Asp338 of the P‐domain (corresponding to Asp347 of calnexin and Asp258 of calreticulin), as mutations of each these residues abolish the interaction [14]. Lys97 of CypB forms salt bridges with Asp338 and Asp332 and hydrogen bonds with the carbonyl of Asp332. Among the many lysine residues of the binding site, only the side chains of Lys9, Lys97, and Lys183 of CypB are involved in the interactions with P‐domain underlying specificity of the binding. Besides interacting with Lys97, the side chain of Asp338 forms an intermolecular hydrogen bond with the side chain of Thr36. Also, the side chain of Met337 at the very tip of the P‐domain inserts between the aliphatic parts of Lys9 and Lys35 of CypB. The absence of a side chain for Gly339 allows for closer approach of the P‐domain to CypB surface [14]. The P‐domains of both calnexin and calreticulin bind CypB with affinity on the order of 10 µm as estimated from NMR studies [14]. This is very similar to the affinities of ERp57 binding to calnexin (*K*_d_ of 6 µm) [55] and to calreticulin (7 µm) [12].

It is very likely that calnexin/calreticulin and CypB interact *in vivo*. CypB and calnexin/calreticulin co‐localize in the ER and are associated with multichaperone ER complexes [62, 63]. The interaction between CypB and calreticulin has been proposed to contribute to ER retention of CypB, which lacks other known ER‐retention signals [64]. The association of glycan‐binding activity with CypB provides a mechanism for the recruitment of PPIase activity in the ER to newly synthesized glycoproteins, such as the C_H_ antibody heavy chain. The heavy‐chain C_H_1 domain possesses three cis‐prolines in its native state, and its folding is markedly accelerated by CypB [65]. Future work is required to test whether monoglucosylation affects the rate of proline isomerization of N‐glycoproteins.

### ERp29

Calnexin and calreticulin have been both found to interact with ERp29 [16, 66, 67], an ER chaperone involved in the folding and secretion of thyroglobulin [68] and collagen [69], polyomavirus entry [70], and dorsal–ventral patterning in Drosophila [71]. Structurally, ERp29 is a dimer composed of an N‐terminal thioredoxin‐like domain and a C‐terminal D‐domain [72, 73, 74, 75, 76] (Fig. 3A). The N‐terminal TRXL domain does not possess disulfide isomerase activity and, unusually for a thioredoxin fold, mediates homodimerization of ERp29. The D‐domain is an all α‐helical domain, which is unique to ERp29.

The NMR studies identified P‐domains of calnexin/calreticulin and the D‐domain of ERp29 as the domains responsible for the ERp29–calnexin/calreticulin interactions [15]. In fact, binding of ERp29 and ERp57 involve the same residues at the tip of the P‐domain from either calnexin or calreticulin [13, 15]. The binding affinity between the ERp29 D‐domain and calnexin P‐domain, or between full‐length ERp29 and calreticulin is in the order of 13 µm measured using NMR and surface plasmon resonance [15, 16].

The ERp29 D‐domain shows an unusual fold where two C‐terminal antiparallel helices are partially solvent‐exposed by extending out from a three‐helix bundle. These solvent‐exposed helices form the binding site for the P‐domain (Fig. 3D). In particular, Arg223 of ERp29 is crucial for the binding as it makes salt bridges with Asp348 of calmegin (Asp258 of calreticulin) and hydrogen bonds with backbone carbonyl of Asp342 (Asp252 of calreticulin). The positively charged Lys204, Lys208, Arg226, and Lys237 of ERp29 are also engaged in polar interactions with the P‐domain. Similar to CypB interactions, the side chain of Met347 at the very tip of the P‐domain binds in a hydrophobic pocket on the ERp29 surface [15].

The D347K mutation in the calnexin P‐domain results in no binding to ERp29. The same mutation was previously shown to abrogate calnexin binding to ERp57 and CypB [14, 77]. Therefore, the same site is responsible for interactions with all three proteins. On the ERp29 side, the R223A, R223E, L227E, and L241K mutations also abolish the binding.

The P‐domains from calnexin, calreticulin, and calmegin are all able to specifically bind the D‐domain of ERp29. The D‐domain of ERp29 is unique in the human genome, but conserved in ERp29 homologs from other species with sequence conservation highest in the P‐domain‐binding residues. Therefore, it is likely that calreticulin/calnexin binding is a conserved ERp29 function across species.

Windbeutel, the *Drosophila* ortholog of ERp29, functions in embryo development through processing of a Golgi sulfotransferase, Pipe [71, 78]. Two regions of Drosophila ERp29 are required for Pipe localization: one in the TRXL domain that mediates binding of denatured thyroglobulin and Pipe, and a second in the D‐domain of previously unclear function [72, 79]. The structural data suggest that the principal function of the D‐domain is calreticulin/calnexin binding. In agreement with that, mutations in the calreticulin/calnexin‐binding site of Drosophila ERp29 block processing of Pipe [79, 80]. In particular, loss of Arg223 blocks both Pipe processing and P‐domain binding. Interestingly, while full‐length human ERp29 cannot replace Drosophila ERp29 for Pipe localization *in vivo*, the D‐domain can be swapped, suggesting a functional conservation of that domain [75]. In another example of functional implication, the calreticulin/calnexin‐binding site is required for the ER retention of the Dictyostelium ERp29 ortholog, which lacks an ER‐retention signal [81].

The dimerization of ERp29 allows for the assembly of larger chaperone complex with two lectin chaperones bound to one ERp29 dimer. While the functional implication of that is currently unclear, this may lead to a tighter binding of multiglycosylated protein substrates. ERp29 dimerization may also play a role in glycosylation‐independent chaperone function by promoting direct binding of nonglycosylated substrates to calreticulin and calnexin.

## Common features of calnexin/calreticulin interactions with partners

Comparison of the crystal structures of P‐domains from calnexin luminal domain [28], calreticulin with partially truncated P‐domain [82], and the P‐domain complexes [14, 15] shows that the structures of P‐domain modules are highly similar despite the intrinsically flexible nature of P‐domains in solution. The rigidity of a module originates from a small hydrophobic core formed by side chains of two tryptophan residues along with lysine followed by a proline residue. The very tip of the P‐domain forms a one‐turn helix. The hydrophobic core and helical turn were observed in the solution structure of the calreticulin P‐domain, confirming that the conformation is formed prior to binding [47]. Thus, the overall flexibility of P‐domains likely arises in the hinge regions between the modules.

A number of residues are highly conserved in the P‐domains. Some of these such as tryptophan and lysine play a structural role, while others are involved in protein binding. Among the latter, a methionine, aspartic acid, and glycine residue at the tip of the P‐domain (the MDG‐binding motif) are absolutely conserved in all family members and are crucial for the ERp57, CypB, and ERp29 binding. The helical turn projects the key binding residues, methionine and following aspartic acid (Met346 and Asp347 in human calnexin, and Met257 and Asp258 in human calreticulin), to their binding partners. The aspartate residue makes key salt bridges with its counterparts, Lys97 of CypB and Arg223 of ERp29. It is tempting to speculate that it interacts with Arg282 of ERp57, but a higher resolution structure is needed to confirm this. Significantly, the calnexin P‐domain D347K mutation abolishes binding to ERp57 [14], CypB, and ERp29, while the homologous aspartic acid is required for calreticulin binding to ERp57 [77]. The side chain of methionine is involved in intermolecular hydrophobic interactions, while the absence of a side chain in glycine residue allows for close packing with the binding partner. It appears that no other residue could be tolerated at this position, explaining the conservation of this glycine in the calnexin/calreticulin protein family. High conservation of these residues (with leucine replacing methionine in CRT3) strongly suggests that this lectin chaperone would also interact with the same binding partners.

Remarkably, the binding sites for the P‐domain are formed from strikingly different structural scaffolds (Fig. 3B–D). The ERp57 site is composed of one helix and two loops; the CypB site consists of loops, while the ERp29‐binding site is all‐helical. Beyond these differences, the common feature is the pronounced positive charge, accounting for the presence of multiple aspartates and glutamates in the P‐domains.

The interactions of calnexin/calreticulin with ERp57, CypB, and ERp29 form a highly interconnected cluster of protein–protein interactions within the ER. The binding affinities to all three proteins are in the same range of 5–15 µm, suggesting no strong preference to any of the partners. While one lectin chaperone can only bind one other associated chaperone, the dynamic nature of interaction likely prevents folding bottlenecks or dead ends. Thus, calreticulin and calnexin appear to act as plurivalent adaptors that recruit other chaperones to assist in different aspects of protein folding, such as disulfide bond formation, proline isomerization, or general chaperone activity. The affinity of the calnexin/calreticulin for monoglucosylated glycans is roughly an order of magnitude higher (0.7 µm) [29], suggesting that the lectin–glycoprotein associations are longer‐lived than the lectin–chaperone associations. This opens a possibility of different chaperones sequentially acting on the same glycoproteins assisting with different aspects of folding.

The sequence conservation of the P‐domains is in sharp contrast with the diversity of binding sites on ERp57, CypB, and ERp29. This suggests that these chaperones became specialized for glycoprotein folding through convergent evolution of their P‐domain‐binding sites. The remarkable versatility of the tip of the P‐domain to interact with different structural scaffolds hints at the existence of other protein partners yet to be discovered.

## Interactions of calreticulin with other ER‐resident proteins

Do calnexin and calreticulin work only as a scaffold by bringing together protein substrates and other chaperones, or do they provide some chaperoning themselves? Because the substrate would be likely positioned between lectin site and another chaperone bound to the tip of P‐domain, it is reasonable to expect some contacts between the protein substrate and the interior side of P‐domain. Indeed, the P‐domain truncation mutants of calreticulin display decreased ability to suppress protein aggregation *in vitro* [82, 83].

Another interesting aspect is the ability of calreticulin and calnexin to bind directly to nonglycosylated hydrophobic peptides with micromolar *K*_d_ [82, 83, 84, 85] or to suppress aggregation of nonglycosylated proteins *in vitro* [82, 86, 87, 88]. This aggregation suppression was mapped to the lectin domain of both calnexin and calreticulin [82, 83]. Consequently, the identification of such peptide binding is of considerable interest. The surfaces overlapping with the lectin site were previously proposed to be binding sites for nonglycosylated substrates [40, 41], but this should be taken with caution. Treatment with monoglucosylated oligosaccharide, which would block the proposed site, does not affect binding of hydrophobic peptides by calreticulin [82].

More recently, a surface distant from lectin site was identified as responsible for *in vitro* binding of nonglycosylated substrates [89]. In particular, two double mutants P19K/I21E and Y22K/F84E of calreticulin do not efficiently suppress aggregation of firefly luciferase and do not bind hydrophobic peptides. The use of these peptide‐binding‐deficient and lectin‐deficient mutants in calreticulin‐negative cells allowed accessing the relative contributions of glycan‐dependent and glycan‐independent in calreticulin function in biogenesis of MHC class I molecules [89]. The conclusion is that the lectin‐based interactions provide the major contribution, whereas the peptide‐binding site has little affect on calreticulin function *in vivo*.

Experiments using T7 phage display system revealed interactions between calreticulin and protein disulfide isomerase‐related (PDIR) protein [90]. The interaction was later confirmed by a mass‐spectrometry study [91]. The affinity of the binding was measured as 16 µm using surface plasmon resonance [90], which would place this interaction into a similar range of affinities with other known calreticulin‐binding partners such as ERp57, CypB, and ERp29. PDIR (also called PDIA5) was originally found in a human placental cDNA library [92]. It is upregulated in mucopolysaccharidoses, diseases caused by defects in degrading glycosaminoglycans [93].

PDIR consists of four thioredoxin‐like domains, but has a unique architecture in PDI family, as it contains an N‐terminal noncatalytic domain followed by three catalytic domains. Crystal structure of the noncatalytic domain identified a conserved positively charged surface, a prime candidate for interacting with the negatively charged P‐domain [94]. Indeed, NMR titrations showed some binding between P‐domain and noncatalytic PDIR domain, but the binding was centered on the hinge region instead of the tip of the P‐domain [94]. It should be noted that the observed interactions were too weak to account for the full affinity. There is still more to learn about the calreticulin‐PDIR binding, and perhaps, future studies would identify other domains of both proteins contributing to this interaction.

Early studies reported interactions between calreticulin and PDI, though the binding was not observed in the presence of Ca^2+^ ions [95]. This work pointed to the P‐domain as a major site of this interaction, but these results may have to be re‐evaluated, as the calreticulin constructs were designed in the absence of structural information at the time. More recent studies tested a panel of seven PDIs (ERp27, ERp29, ERp44, ERp46, ERp57, PDI, and PDIp) for calreticulin interactions by surface plasmon resonance and only identified ERp29 and ERp57 as calreticulin‐binding proteins [16].

There are intriguing similarities in glycoproteins processing by calnexin cycle and ER‐associated degradation (ERAD) machineries. They both heavily rely on the state of the glycan, which is recognized and captured by ER lectins, CNX/CRT and UGGT in calnexin cycle and the ER degradation‐enhancing α‐mannosidase‐like proteins (EDEMs) in ERAD. Both systems also display functional and specific interactions with a number of PDIs, where the latter often responsible for reduction and/or reshuffling disulfides in glycoprotein clients. Those include CNX/CRT‐ERp57 and UGGT‐Sep15 pairings in calnexin cycle, while EDEM1‐ERdj5 and EDEM2‐TXNDC11 display reminiscent functional cooperativity in ERAD [96, 97, 98].

Does glucosidase II interact with calnexin/calreticulin, or it gets recruited via another member of calnexin cycle? How do they compete for monoglucosylated proteins? This has important implications on the rate with which glycoproteins escape from calnexin cycle. One study showed the preference of glucosidase II for folded versus misfolded monoglucosylated substrates when in the presence of calreticulin but not on its own [99], but there is still much to learn on their interplay. Future studies will likely uncover more calnexin/calreticulin interactions with other ER‐resident proteins, a result of comprehensive folding machinery in the ER.

## Structure of UGGT

N‐glycoproteins that are difficult to fold undergo multiple rounds of folding with assistance of ER lectin chaperones. By reglucosylating misfolded proteins, UGGT plays the role of a checkpoint allowing misfolded proteins to rebind to the lectin chaperones and preventing their exit from the ER. UGGT expression is elevated upon ER stress and is a part of unfolded protein response [10]. UGGT also controls the loading of peptide antigens onto major immunological molecules, T‐cell receptor, and the major histocompatibility complex [17, 18, 19, 20]. Most vertebrates possess two homologous genes UGGT1 and UGGT2. UGGT2 shares significant sequence identity (55%) to UGGT1 but does not display comparable reglucosylation activity on certain substrates [11]. More recently, UGGT2 was shown to possess enzymatic activity using synthetic substrates [102, 103]. It is very likely that UGGT1 and UGGT2 evolved to have different clients in glycoprotein folding pathway. UGGT2 has been recently proposed to serve as a folding checkpoint for a distinct set of yet‐to‐be‐identified misfolded glycoproteins [14].

Mammalian UGGTs are approximately 1500‐residue proteins, where the N‐terminal ~ 1200 residues are responsible for sensing misfolded substrates and the C‐terminal ~ 300 residues harbor a glucosyltransferase 24 family (GT24) A‐type catalytic domain (Fig. 4A). For a long time, multiple efforts to structurally characterize UGGT were unsuccessful, with only the structure of one of the domains determined in 2014 [15]. Finally, there was a breakthrough in 2017 with several laboratories reporting UGGT structures by X‐ray crystallography, electron microscopy, and small‐angle X‐ray scattering [22, 23, 24]. We now have a comprehensive view of the structure of UGGT. All crystal structures have been done on UGGT from thermophilic fungi, which possess a single UGGT gene. Nevertheless, the structural conclusions should be applicable to both UGGT1 and UGGT2 in vertebrates given high sequence identity between UGGT1 and UGGT2. The crystal structures show UGGT forms a saddle‐like shape with a large central cavity (Fig. 4B) [22, 25]. The shape is consistent with the low‐resolution EM structures and with molecular envelope obtained in solution using SAXS data [22, 23, 24].

The structure consists of four N‐terminal αβ‐sandwich domains, followed by a saddle‐shaped pair of β‐sandwich domains that seat the catalytic domain (Fig. 4B,C). Overall, the N‐terminal domains of UGGT are very unusual and structurally more similar to DsbA than to PDIs (Fig. 4C,D). They were assigned their own families by Pfam database of structural folds: Thioredoxin_12 (PF18400), Thioredoxin_13 (PF18401), Thioredoxin_14 (PF18402), and Thioredoxin_15 (PF18403) for UGGT domains 1, 2, 3, and 4, respectively. Rather confusingly, they were termed thioredoxin‐like (TRXL) domains despite their significant deviation from the canonical PDI‐like TRXL fold (PF13848) and obvious similarity to DsbA fold (PF01323). However, for consistency with the previous UGGT literature, we are referring to the αβ‐sandwich UGGT domains as TRXL1, TRXL2, TRXL3, and TRXL4 in this review.

While the first αβ‐sandwich UGGT domain resembles DsbA (Fig. 4D), the order of secondary structure elements is different. In DsbA (***βαβ−αααα−αββα***), the helical subdomain arises from residues inserted into the middle of the thioredoxin fold, while in the first UGGT domain (***αααα−βαβ–αββα***), the helical elements precede the thioredoxin fold. Even more striking, the TRXL4 and β‐sandwich domains are folded with discontinuous regions of the primary sequence (Fig. 4A). This complex topology is largely responsible for earlier difficulties in predicting UGGT structural domains.

The similarity of domains of UGGT to DsbA fold raises the question of the origin of UGGT. While it is generally assumed that many PDIs originated via gene duplication of TRXL domains, it does not appear to apply to UGGT. TRXL2 and TRXL3 are most similar among UGGT domains, but even they possess significant differences (Fig. 4C).

High‐resolution structures of UGGT catalytic domain were determined in complex with UDP‐glucose and UDP [24]. The structure shows significant fold similarity with GT8 family of glycosyltransferases [16]. UDP‐glucose and the catalytically important calcium ion are buried in the active site with two aspartates from the invariant DxD motif coordinating Ca^2+^ (Fig. 4E). One of the helices (residues 1325–1344 corresponding to residues 1389–1408 of human UGGT1) on the edge of the active site is significantly distorted, with the place of distortion creating a flat cavity leading to the UDP‐glucose hydrolysis site. This is similar to the position of substrate in another member of GT8 family, a galactosyltransferase LgtC [16] (Fig. 4E). Thus, it is a very likely location of the glycan entrance in UGGT. Vicinity of the active site contains a number of small patches of hydrophobic residues such as Phe1333, Gly1337, Tyr1338, and Trp1339 (Phe1397, Gly1401, Tyr1402, and Trp1403 in human UGGT1). Another patch consists of Phe1331, Pro1432, and Leu1433 (Tyr1395, Pro1496, and Met1497 in human UGGT1). This may explain previous results of UGGT1 catalytic domain interacting with synthetic hydrophobic aglycon [17].

## Mechanism of action of UGGT

Despite the recent breakthrough in UGGT structural characterization, the mechanism of its action is still not clear. The full‐length UGGT structures showed only a limited range of mobility with the catalytic domain fixed to the β‐sandwich domain, while the main source of mobility originates from TRXL3 and especially TRXL2 domain (Fig. 4F) [22]. Comparison of the full‐length *Ch. termophilum* UGGT structures with the catalytic domain‐deleted fragment of UGGT from *Th. dupontii* [24] similarly shows large shifts in the positions of TRXL2 and TRXL3 domains, while the relative positions of the TRXL1, TRXL4, and β‐rich domains are preserved. This suggests that TRXL1, TRXL4, and β‐sandwich domains comprise a rigid scaffold, while the TRXL2, TRXL3, and catalytic domains account for the ability of UGGT to act on protein substrates of differing sizes and shapes. In agreement with this, UGGT activity was impaired when mobility of its N‐terminal domains was limited using engineered interdomain disulfide bonds [22]. Thus, flexibility appears to be important for UGGT activity and versatility toward numerous substrates in the cell.

Because of their influence on the size of the saddle, the TRXL2 and TRXL3 domains are expected to be partially responsible for recognizing misfolded stretches of protein substrates. Multiple TRXL domains were shown to convey binding of hydrophobic stretches starting from protein disulfide isomerase (PDI) itself to other members of PDI family [2, 94, 108]. Most likely, the cavity‐faced surfaces of UGGT TRXL2 and TRXL3 domains participate in recognition of misfolded substrates. The important role of TRXL2 in substrate reglucosylation has been recently supported by UGGT deletion mutagenesis and molecular dynamics simulations [25]. Future mutagenesis studies should confirm the substrate‐binding surfaces.

There is still a lack of clarity in the mode of catalytic domain involvement. Early theories proposed a great deal of mobility between the N‐terminal part and catalytic domain, while the full‐length UGGT structures invariably showed the catalytic domain firmly entrenched in the β‐sandwich surface [22]. At the same time, negative‐stain EM and SAXS data implied significant movements of the catalytic domain in solution [24], but alternative interpretation is also possible [25]. Notably, the ability of catalytic domain to be stable in solution independently from the rest of UGGT is supported by crystal structures of individual catalytic domain [24]. Perhaps, the release of catalytic domain from the β‐sandwich domain may be facilitated by binding to UDP‐glucose and/or protein substrate. This contradiction can be resolved by permanently tethering catalytic domain to the β‐sandwich domains via engineered disulfide bonds and testing the mutant for activity.

## UGGT‐Sep15 interactions

UGGT1 binds with high affinity (K_d_ of 20 nM) to ER oxidoreductase Sep15 [19]. Sep15 (also called 15‐kDa selenoprotein or selenoprotein F) is a member of small family of selenoproteins found in the ER [110]. Sep15 lacks a typical ER‐retrieval signal suggesting that it is maintained in the ER via a different mechanism, most likely through high‐affinity binding to UGGT1. Supporting that hypothesis, the entire pool of Sep15 was shown to be bound to UGGT1, while UGGT1 occurs in both Sep15‐bound and free states [111].

Structurally, Sep15 consists of two domains, a ~ 50‐residue cysteine‐rich N‐terminal domain followed by a Sep15/SelM redox domain (Pfam Sep15_SelM family PF08806). The Sep15 redox domain contains selenocysteine (U), which is separated from cysteine by a single residue in Sep15 catalytic motif (CxU). This is a deviation from typical oxidoreductases, including PDIs, which possess CxxC catalytic motif. The NMR structure of this redox domain revealed significant differences from thioredoxin [112]. In particular, the structure contains a four‐stranded β‐sheet with α‐helices on only one side (Fig. 4D). In comparison with a typical thioredoxin fold, the structure is missing two helices so that one side of the β‐sheet is solvent‐exposed. This surface presents several hydrophobic residues, which could potentially interact with misfolded substrates. It is also possible that this surface is used for binding UGGT1 or intramolecular contacts with the N‐terminal domain of Sep15. The fold is also missing an N‐terminal β‐strand that is usually found in thioredoxin‐like domains in the PDI family [2]. Thus, Sep15 represents a simplified topology of the redox domain with the βαβββα organization as compared to a most typical βαβαβαββα thioredoxin‐like fold in PDI family. Curiously, unlike thioredoxin‐like domains, the catalytic motif of Sep15 is located in a loop rather than at the N terminus of an α‐helix.

Why does Sep15 contain selenocysteine in place of one of the cysteines in its active site? Selenocysteine likely modifies the redox potential affecting the potency of its oxidoreductase activity; however, it does not appear to be a requirement for Sep15 function as the *Drosophila* ortholog possesses a cysteine as opposed to selenocysteine. The redox potential of *Drosophila* Sep15 is −225 mV [112], which lies between the potentials of the protein disulfide oxidase PDIA1 (−175 mV) [113] and thioredoxin (−270 mV) [114]. This suggests that Sep15 is likely involved in the reduction or isomerization of disulfide bonds (rather than their formation).

Sep15 possesses a distinct cysteine‐rich N‐terminal domain, which is responsible for binding to UGGT1 [19]. Six invariantly conserved cysteines were shown to be critical for the interaction. As the structure of this domain is still unknown, it is currently unclear whether these cysteines actually contact UGGT1 or play a structural role. A structure of UGGT1 in complex with Sep15 will provide important mechanistic insights into Sep15‐UGGT1 cooperativity in protein folding.

What is the role of Sep15 in the function of UGGT1 and the calnexin/calreticulin cycle in general? Previous studies showed enhancement of UGGT1 and UGGT2 activities upon binding to Sep15 [102, 103]. Recent results suggest that Sep15 prevents secretion of disulfide‐rich glycoproteins with incorrectly formed disulfides to Golgi providing additional step of quality control in the ER [115]. It is plausible that Sep15 enhances UGGT activity via reduction in incorrect intramolecular/intermolecular disulfides in misfolded UGGT substrates, thus enabling easier access of glycan to the UGGT active site. This is reminiscent of the EDEM‐ERdj5 cooperation [96].

## Future directions

Recent years have seen new exciting developments in structural understanding of folding pathways of glycoproteins in the ER and brought new potential members of the calnexin cycle into the light. Despite this progress, many questions still remain unanswered. The molecular details of UGGT action are still not fully understood. What is the basis of Sep15 involvement in UGGT function? Future studies of UGGT complexes with Sep15 and substrates would clarify many of these aspects.

On the calnexin/calreticulin side, recent insights provided an exciting view of structural organization of these proteins and how they recruit their helpers assisting in folding N‐glycosylated substrates. Calnexin and calreticulin have been traditionally viewed as chaperones, but in light of recent studies they rather appear to function as scaffolds. We now have a much better understanding of their scaffolding function, where the P‐domain works as a long flexible arm that recruits a folding assistant and brings it to a glycosylated substrate captured via the lectin domain. It also became apparent that this process is much more complex than originally thought and involves multiple folding assistants besides ERp57. Because glycan‐based interactions are approximately 10‐fold stronger resulting in a longer lifetime of the bound state, calnexin/calreticulin likely shuffles through multiple chaperones assisting with different aspects of protein folding of any single substrate. Based on recent developments, it would not be surprising if additional chaperone partners of calnexin/calreticulin will be discovered in future years. It will also be interesting to see whether there are other ways in which calnexin/calreticulin can bind chaperones (such as PDIR) and whether this could lead to the formation of multichaperone complexes to assist with folding of specific substrates.

An important unanswered question in the field is the interplay of calnexin/calreticulin and UGGT with glucosidase II. Are activities of lectin chaperones, glucosidase II, and UGGT coordinated in any way? How does glucosidase II get recruited into calnexin cycle and how it competes with calnexin/calreticulin for monoglucosylated substrates? There is still much to learn about calnexin cycle pathway, and the future years will undoubtedly bring us more exciting discoveries.

## Conflicts of interest

The authors declare no conflict of interest.

## Author contributions

GK and KG wrote the review.

## References

[1] Ellgaard L , McCaul N , Chatsisvili A & Braakman I (2016) Co‐ and post‐translational protein folding in the ER. Traffic 17, 615–638. 26947578 10.1111/tra.12392

[2] Kozlov G , Maattanen P , Thomas DY & Gehring K (2010) A structural overview of the PDI family of proteins. FEBS J 277, 3924–3936. 20796029 10.1111/j.1742-4658.2010.07793.x

[3] Ellgaard L & Ruddock LW (2005) The human protein disulphide isomerase family: substrate interactions and functional properties. EMBO Rep 6, 28–32. 15643448 10.1038/sj.embor.7400311 PMC1299221

[4] Hatahet F & Ruddock LW (2009) Protein disulfide isomerase: a critical evaluation of its function in disulfide bond formation. Antioxid Redox Signal 11, 2807–2850. 19476414 10.1089/ars.2009.2466

[5] Hammond C , Braakman I & Helenius A (1994) Role of N‐linked oligosaccharide recognition, glucose trimming, and calnexin in glycoprotein folding and quality control. Proc Natl Acad Sci U S A 91, 913–917. 8302866 10.1073/pnas.91.3.913 PMC521423

[6] Helenius A & Aebi M (2004) Roles of N‐linked glycans in the endoplasmic reticulum. Annu Rev Biochem 73, 1019–1049. 15189166 10.1146/annurev.biochem.73.011303.073752

[7] Tannous A , Pisoni GB , Hebert DN & Molinari M (2015) N‐linked sugar‐regulated protein folding and quality control in the ER. Semin Cell Dev Biol 41, 79–89. 25534658 10.1016/j.semcdb.2014.12.001 PMC4474783

[8] Lamriben L , Graham JB , Adams BM & Hebert DN (2016) N‐Glycan‐based ER molecular chaperone and protein quality control system: The Calnexin Binding Cycle. Traffic 17, 308–326. 26676362 10.1111/tra.12358 PMC4805476

[9] Zapun A , Petrescu SM , Rudd PM , Dwek RA , Thomas DY & Bergeron JJ (1997) Conformation‐independent binding of monoglucosylated ribonuclease B to calnexin. Cell 88, 29–38. 9019402 10.1016/s0092-8674(00)81855-3

[10] Ou WJ , Cameron PH , Thomas DY & Bergeron JJ (1993) Association of folding intermediates of glycoproteins with calnexin during protein maturation. Nature 364, 771–776. 8102790 10.1038/364771a0

[11] Zapun A , Darby NJ , Tessier DC , Michalak M , Bergeron JJ & Thomas DY (1998) Enhanced catalysis of ribonuclease B folding by the interaction of calnexin or calreticulin with ERp57. J Biol Chem 273, 6009–6012. 9497314 10.1074/jbc.273.11.6009

[12] Frickel EM , Riek R , Jelesarov I , Helenius A , Wuthrich K & Ellgaard L (2002) TROSY‐NMR reveals interaction between ERp57 and the tip of the calreticulin P‐domain. Proc Natl Acad Sci U S A 99, 1954–1959. 11842220 10.1073/pnas.042699099 PMC122301

[13] Pollock S , Kozlov G , Pelletier MF , Trempe JF , Jansen G , Sitnikov D , Bergeron JJ , Gehring K , Ekiel I & Thomas DY (2004) Specific interaction of ERp57 and calnexin determined by NMR spectroscopy and an ER two‐hybrid system. EMBO J 23, 1020–1029. 14988724 10.1038/sj.emboj.7600119 PMC380975

[14] Kozlov G , Bastos‐Aristizabal S , Maattanen P , Rosenauer A , Zheng F , Killikelly A , Trempe JF , Thomas DY & Gehring K (2010) Structural basis of cyclophilin B binding by the calnexin/calreticulin P‐domain. J Biol Chem 285, 35551–35557. 20801878 10.1074/jbc.M110.160101 PMC2975179

[15] Kozlov G , Munoz‐Escobar J , Castro K & Gehring K (2017) Mapping the ER interactome: The P domains of calnexin and calreticulin as plurivalent adapters for foldases and chaperones. Structure 25, 1415–1422.e1413. 28877505 10.1016/j.str.2017.07.010

[16] Sakono M , Seko A , Takeda Y & Ito Y (2014) PDI family protein ERp29 forms 1:1 complex with lectin chaperone calreticulin. Biochem Biophys Res Commun 452, 27–31. 25130463 10.1016/j.bbrc.2014.08.041

[17] Blum JS , Wearsch PA & Cresswell P (2013) Pathways of antigen processing. Annu Rev Immunol 31, 443–473. 23298205 10.1146/annurev-immunol-032712-095910 PMC4026165

[18] Neerincx A , Hermann C , Antrobus R , van Hateren A , Cao H , Trautwein N , Stevanovic S , Elliott T , Deane JE & Boyle LH (2017) TAPBPR bridges UDP‐glucose:glycoprotein glucosyltransferase 1 onto MHC class I to provide quality control in the antigen presentation pathway. eLife 6, e23049. 28425917 10.7554/eLife.23049 PMC5441866

[19] Zhang W , Wearsch PA , Zhu Y , Leonhardt RM & Cresswell P (2011) A role for UDP‐glucose glycoprotein glucosyltransferase in expression and quality control of MHC class I molecules. Proc Natl Acad Sci U S A 108, 4956–4961. 21383159 10.1073/pnas.1102527108 PMC3064381

[20] Gardner TG & Kearse KP (1999) Modification of the T cell antigen receptor (TCR) complex by UDP‐glucose:glycoprotein glucosyltransferase. TCR folding is finalized convergent with formation of alpha beta delta epsilon gamma epsilon complexes. J Biol Chem 274, 14094–14099. 10318825 10.1074/jbc.274.20.14094

[21] Blees A , Januliene D , Hofmann T , Koller N , Schmidt C , Trowitzsch S , Moeller A & Tampe R (2017) Structure of the human MHC‐I peptide‐loading complex. Nature 551, 525–528. 29107940 10.1038/nature24627

[22] Roversi P , Marti L , Caputo AT , Alonzi DS , Hill JC , Dent KC , Kumar A , Levasseur MD , Lia A , Waksman T *et al* (2017) Interdomain conformational flexibility underpins the activity of UGGT, the eukaryotic glycoprotein secretion checkpoint. Proc Natl Acad Sci U S A 114, 8544–8549. 28739903 10.1073/pnas.1703682114 PMC5559018

[23] Calles‐Garcia D , Yang M , Soya N , Melero R , Menade M , Ito Y , Vargas J , Lukacs GL , Kollman JM , Kozlov G & *et al* (2017) Single‐particle electron microscopy structure of UDP‐glucose:glycoprotein glucosyltransferase suggests a selectivity mechanism for misfolded proteins. J Biol Chem 292, 11499–11507. 28490633 10.1074/jbc.M117.789495 PMC5500813

[24] Satoh T , Song C , Zhu T , Toshimori T , Murata K , Hayashi Y , Kamikubo H , Uchihashi T & Kato K (2017) Visualisation of a flexible modular structure of the ER folding‐sensor enzyme UGGT. Sci Rep 7, 12142. 28939828 10.1038/s41598-017-12283-w PMC5610325

[25] Modenutti CP , Blanco Capurro JI , Ibba R , Vasiljevic S , Hensen M , Alonzi DS , Chandran AV , Hill JC , Rushton J , Kumar A *et al* (2019) Clamping, bending, and twisting inter‐domain motions in the misfold‐recognising portion of UDP‐glucose:glycoprotein glucosyl‐transferase. BioRxiv 888438 [Preprint]. 10.1016/j.str.2020.11.017 PMC8024514 33352114

[26] Satoh T & Kato K (2018) Structural aspects of ER glycoprotein quality‐control system mediated by glucose tagging. Adv Exp Med Biol 1104, 149–169. 30484248 10.1007/978-981-13-2158-0_8

[27] Caputo AT , Alonzi DS , Kiappes JL , Struwe WB , Cross A , Basu S , Darlot B , Roversi P & Zitzmann N (2018) Structural insights into the broad‐spectrum antiviral target endoplasmic reticulum alpha‐glucosidase II. Adv Exp Med Biol 1062, 265–276. 29845539 10.1007/978-981-10-8727-1_19

[28] Schrag JD , Bergeron JJ , Li Y , Borisova S , Hahn M , Thomas DY & Cygler M (2001) The Structure of calnexin, an ER chaperone involved in quality control of protein folding. Mol Cell 8, 633–644. 11583625 10.1016/s1097-2765(01)00318-5

[29] Kozlov G , Pocanschi CL , Rosenauer A , Bastos‐Aristizabal S , Gorelik A , Williams DB & Gehring K (2010) Structural basis of carbohydrate recognition by calreticulin. J Biol Chem 285, 38612–38620. 20880849 10.1074/jbc.M110.168294 PMC2992293

[30] Villamil Giraldo AM , Lopez Medus M , Gonzalez Lebrero M , Pagano RS , Labriola CA , Landolfo L , Delfino JM , Parodi AJ & Caramelo JJ (2010) The structure of calreticulin C‐terminal domain is modulated by physiological variations of calcium concentration. J Biol Chem 285, 4544–4553. 20018892 10.1074/jbc.M109.034512 PMC2836059

[31] Kapoor M , Ellgaard L , Gopalakrishnapai J , Schirra C , Gemma E , Oscarson S , Helenius A & Surolia A (2004) Mutational analysis provides molecular insight into the carbohydrate‐binding region of calreticulin: pivotal roles of tyrosine‐109 and aspartate‐135 in carbohydrate recognition. Biochemistry 43, 97–106. 14705935 10.1021/bi0355286

[32] Thomson SP & Williams DB (2005) Delineation of the lectin site of the molecular chaperone calreticulin. Cell Stress Chaperones 10, 242–251. 16184769 10.1379/CSC-126.1 PMC1226022

[33] Kapoor M , Srinivas H , Kandiah E , Gemma E , Ellgaard L , Oscarson S , Helenius A & Surolia A (2003) Interactions of substrate with calreticulin, an endoplasmic reticulum chaperone. J Biol Chem 278, 6194–6200. 12464625 10.1074/jbc.M209132200

[34] Danilczyk UG , Cohen‐Doyle MF & Williams DB (2000) Functional relationship between calreticulin, calnexin, and the endoplasmic reticulum luminal domain of calnexin. J Biol Chem 275, 13089–13097. 10777614 10.1074/jbc.275.17.13089

[35] Peterson JR , Ora A , Van PN & Helenius A (1995) Transient, lectin‐like association of calreticulin with folding intermediates of cellular and viral glycoproteins. Mol Biol Cell 6, 1173–1184. 8534914 10.1091/mbc.6.9.1173 PMC301275

[36] Pieren M , Galli C , Denzel A & Molinari M (2005) The use of calnexin and calreticulin by cellular and viral glycoproteins. J Biol Chem 280, 28265–28271. 15951445 10.1074/jbc.M501020200

[37] Wada I , Imai S , Kai M , Sakane F & Kanoh H (1995) Chaperone function of calreticulin when expressed in the endoplasmic reticulum as the membrane‐anchored and soluble forms. J Biol Chem 270, 20298–20304. 7657600 10.1074/jbc.270.35.20298

[38] Hebert DN , Zhang JX , Chen W , Foellmer B & Helenius A (1997) The number and location of glycans on influenza hemagglutinin determine folding and association with calnexin and calreticulin. J Cell Biol 139, 613–623. 9348279 10.1083/jcb.139.3.613 PMC2141715

[39] Vassilakos A , Michalak M , Lehrman MA & Williams DB (1998) Oligosaccharide binding characteristics of the molecular chaperones calnexin and calreticulin. Biochemistry 37, 3480–3490. 9521669 10.1021/bi972465g

[40] Martin V , Groenendyk J , Steiner SS , Guo L , Dabrowska M , Parker JM , Muller‐Esterl W , Opas M & Michalak M (2006) Identification by mutational analysis of amino acid residues essential in the chaperone function of calreticulin. J Biol Chem 281, 2338–2346. 16291754 10.1074/jbc.M508302200

[41] Chouquet A , Paidassi H , Ling WL , Frachet P , Houen G , Arlaud GJ & Gaboriaud C (2011) X‐ray structure of the human calreticulin globular domain reveals a peptide‐binding area and suggests a multi‐molecular mechanism. PLoS ONE 6, e17886. 21423620 10.1371/journal.pone.0017886 PMC3057994

[42] Baksh S & Michalak M (1991) Expression of calreticulin in *Escherichia coli* and identification of its Ca2+ binding domains. J Biol Chem 266, 21458–21465. 1939178

[43] Nakamura K , Zuppini A , Arnaudeau S , Lynch J , Ahsan I , Krause R , Papp S , De Smedt H , Parys JB , Muller‐Esterl W *et al* (2001) Functional specialization of calreticulin domains. J Cell Biol 154, 961–972. 11524434 10.1083/jcb.200102073 PMC2196195

[44] Tjoelker LW , Seyfried CE , Eddy RL Jr , Byers MG , Shows TB , Calderon J , Schreiber RB & Gray PW (1994) Human, mouse, and rat calnexin cDNA cloning: identification of potential calcium binding motifs and gene localization to human chromosome 5. Biochemistry 33, 3229–3236. 8136357 10.1021/bi00177a013

[45] Wijeyesakere SJ , Bedi SK , Huynh D & Raghavan M (2016) The C‐Terminal acidic region of calreticulin mediates phosphatidylserine binding and apoptotic cell phagocytosis. J Immunol 196, 3896–3909. 27036911 10.4049/jimmunol.1502122 PMC5222549

[46] Wijeyesakere SJ , Gafni AA & Raghavan M (2011) Calreticulin is a thermostable protein with distinct structural responses to different divalent cation environments. J Biol Chem 286, 8771–8785. 21177861 10.1074/jbc.M110.169193 PMC3058961

[47] Ellgaard L , Riek R , Herrmann T , Guntert P , Braun D , Helenius A & Wuthrich K (2001) NMR structure of the calreticulin P‐domain. Proc Natl Acad Sci U S A 98, 3133–3138. 11248044 10.1073/pnas.051630098 PMC30619

[48] Raghavan M , Wijeyesakere SJ , Peters LR & Del Cid N (2013) Calreticulin in the immune system: ins and outs. Trends Immunol 34, 13–21. 22959412 10.1016/j.it.2012.08.002 PMC4117402

[49] Oliver JD , van der Wal FJ , Bulleid NJ & High S (1997) Interaction of the thiol‐dependent reductase ERp57 with nascent glycoproteins. Science 275, 86–88. 8974399 10.1126/science.275.5296.86

[50] Bourdi M , Demady D , Martin JL , Jabbour SK , Martin BM , George JW & Pohl LR (1995) cDNA cloning and baculovirus expression of the human liver endoplasmic reticulum P58: characterization as a protein disulfide isomerase isoform, but not as a protease or a carnitine acyltransferase. Arch Biochem Biophys 323, 397–403. 7487104 10.1006/abbi.1995.0060

[51] Hirano N , Shibasaki F , Sakai R , Tanaka T , Nishida J , Yazaki Y , Takenawa T & Hirai H (1995) Molecular cloning of the human glucose‐regulated protein ERp57/GRP58, a thiol‐dependent reductase. Identification of its secretory form and inducible expression by the oncogenic transformation. Eur J Biochem 234, 336–342. 8529662 10.1111/j.1432-1033.1995.336_c.x

[52] Srivastava SP , Fuchs JA & Holtzman JL (1993) The reported cDNA sequence for phospholipase C alpha encodes protein disulfide isomerase, isozyme Q‐2 and not phospholipase‐C. Biochem Biophys Res Commun 193, 971–978. 8391814 10.1006/bbrc.1993.1720

[53] Oliver JD , Roderick HL , Llewellyn DH & High S (1999) ERp57 functions as a subunit of specific complexes formed with the ER lectins calreticulin and calnexin. Mol Biol Cell 10, 2573–2582. 10436013 10.1091/mbc.10.8.2573 PMC25489

[54] Leach MR , Cohen‐Doyle MF , Thomas DY & Williams DB (2002) Localization of the lectin, ERp57 binding, and polypeptide binding sites of calnexin and calreticulin. J Biol Chem 277, 29686–29697. 12052826 10.1074/jbc.M202405200

[55] Kozlov G , Maattanen P , Schrag JD , Pollock S , Cygler M , Nagar B , Thomas DY & Gehring K (2006) Crystal structure of the bb' domains of the protein disulfide isomerase ERp57. Structure 14, 1331–1339. 16905107 10.1016/j.str.2006.06.019

[56] Dong G , Wearsch PA , Peaper DR , Cresswell P & Reinisch KM (2009) Insights into MHC class I peptide loading from the structure of the tapasin‐ERp57 thiol oxidoreductase heterodimer. Immunity 30, 21–32. 19119025 10.1016/j.immuni.2008.10.018 PMC2650231

[57] Price ER , Zydowsky LD , Jin MJ , Baker CH , McKeon FD & Walsh CT (1991) Human cyclophilin B: a second cyclophilin gene encodes a peptidyl‐prolyl isomerase with a signal sequence. Proc Natl Acad Sci U S A 88, 1903–1907. 2000394 10.1073/pnas.88.5.1903 PMC51134

[58] Hasel KW , Glass JR , Godbout M & Sutcliffe JG (1991) An endoplasmic reticulum‐specific cyclophilin. Mol Cell Biol 11, 3484–3491. 1710767 10.1128/mcb.11.7.3484-3491.1991 PMC361082

[59] Steinmann B , Bruckner P & Superti‐Furga A (1991) Cyclosporin A slows collagen triple‐helix formation in vivo: indirect evidence for a physiologic role of peptidyl‐prolyl cis‐trans‐isomerase. J Biol Chem 266, 1299–1303. 1985948

[60] Lodish HF & Kong N (1991) Cyclosporin A inhibits an initial step in folding of transferrin within the endoplasmic reticulum. J Biol Chem 266, 14835–14838. 1714445

[61] Kim J , Choi TG , Ding Y , Kim Y , Ha KS , Lee KH , Kang I , Ha J , Kaufman RJ , Lee J *et al* (2008) Overexpressed cyclophilin B suppresses apoptosis associated with ROS and Ca2+ homeostasis after ER stress. J Cell Sci 121, 3636–3648. 18946027 10.1242/jcs.028654 PMC2735721

[62] Meunier L , Usherwood YK , Chung KT & Hendershot LM (2002) A subset of chaperones and folding enzymes form multiprotein complexes in endoplasmic reticulum to bind nascent proteins. Mol Biol Cell 13, 4456–4469. 12475965 10.1091/mbc.E02-05-0311 PMC138646

[63] Zhang J & Herscovitz H (2003) Nascent lipidated apolipoprotein B is transported to the Golgi as an incompletely folded intermediate as probed by its association with network of endoplasmic reticulum molecular chaperones, GRP94, ERp72, BiP, calreticulin, and cyclophilin B. J Biol Chem 278, 7459–7468. 12397072 10.1074/jbc.M207976200

[64] Arber S , Krause KH & Caroni P (1992) s‐cyclophilin is retained intracellularly via a unique COOH‐terminal sequence and colocalizes with the calcium storage protein calreticulin. J Cell Biol 116, 113–125. 1530944 10.1083/jcb.116.1.113 PMC2289259

[65] Feige MJ , Groscurth S , Marcinowski M , Shimizu Y , Kessler H , Hendershot LM & Buchner J (2009) An unfolded CH1 domain controls the assembly and secretion of IgG antibodies. Mol Cell 34, 569–579. 19524537 10.1016/j.molcel.2009.04.028 PMC2908990

[66] Nakao H , Seko A , Ito Y & Sakono M (2017) PDI family protein ERp29 recognizes P‐domain of molecular chaperone calnexin. Biochem Biophys Res Commun 487, 763–767. 28456374 10.1016/j.bbrc.2017.04.139

[67] Park S , You KH , Shong M , Goo TW , Yun EY , Kang SW & Kwon OY (2005) Overexpression of ERp29 in the thyrocytes of FRTL‐5 cells. Mol Biol Rep 32, 7–13. 15865205 10.1007/s11033-004-3069-3

[68] Baryshev M , Sargsyan E & Mkrtchian S (2006) ERp29 is an essential endoplasmic reticulum factor regulating secretion of thyroglobulin. Biochem Biophys Res Commun 340, 617–624. 16380091 10.1016/j.bbrc.2005.12.052

[69] DiChiara AS , Taylor RJ , Wong MY , Doan ND , Rosario AM & Shoulders MD (2016) Mapping and exploring the collagen‐I proteostasis network. ACS Chem Biol 11, 1408–1421. 26848503 10.1021/acschembio.5b01083 PMC4910512

[70] Magnuson B , Rainey EK , Benjamin T , Baryshev M , Mkrtchian S & Tsai B (2005) ERp29 triggers a conformational change in polyomavirus to stimulate membrane binding. Mol Cell 20, 289–300. 16246730 10.1016/j.molcel.2005.08.034

[71] Konsolaki M & Schupbach T (1998) windbeutel, a gene required for dorsoventral patterning in Drosophila, encodes a protein that has homologies to vertebrate proteins of the endoplasmic reticulum. Genes Dev 12, 120–131. 9420336 10.1101/gad.12.1.120 PMC316405

[72] Barak NN , Neumann P , Sevvana M , Schutkowski M , Naumann K , Malesevic M , Reichardt H , Fischer G , Stubbs MT & Ferrari DM (2009) Crystal structure and functional analysis of the protein disulfide isomerase‐related protein ERp29. J Mol Biol 385, 1630–1642. 19084538 10.1016/j.jmb.2008.11.052

[73] Ferrari DM , Van Nguyen P , Kratzin HD & Soling HD (1998) ERp28, a human endoplasmic‐reticulum‐lumenal protein, is a member of the protein disulfide isomerase family but lacks a CXXC thioredoxin‐box motif. Eur J Biochem 255, 570–579. 9738895 10.1046/j.1432-1327.1998.2550570.x

[74] Liepinsh E , Baryshev M , Sharipo A , Ingelman‐Sundberg M , Otting G & Mkrtchian S (2001) Thioredoxin fold as homodimerization module in the putative chaperone ERp29: NMR structures of the domains and experimental model of the 51 kDa dimer. Structure 9, 457–471. 11435111 10.1016/s0969-2126(01)00607-4

[75] Ma Q , Guo C , Barnewitz K , Sheldrick GM , Soling HD , Uson I & Ferrari DM (2003) Crystal structure and functional analysis of Drosophila Wind, a protein‐disulfide isomerase‐related protein. J Biol Chem 278, 44600–44607. 12941941 10.1074/jbc.M307966200

[76] Mkrtchian S , Baryshev M , Matvijenko O , Sharipo A , Sandalova T , Schneider G & Ingelman‐Sundberg M (1998) Oligomerization properties of ERp29, an endoplasmic reticulum stress protein. FEBS Lett 431, 322–326. 9714535 10.1016/s0014-5793(98)00786-8

[77] Zhang Y , Kozlov G , Pocanschi CL , Brockmeier U , Ireland BS , Maattanen P , Howe C , Elliott T , Gehring K & Williams DB (2009) ERp57 does not require interactions with Calnexin and Calreticulin to promote assembly of Class I histocompatibility molecules, and it enhances peptide loading independently of its redox activity. J Biol Chem 284, 10160–10173. 19196713 10.1074/jbc.M808356200 PMC2665070

[78] Sen J , Goltz JS , Konsolaki M , Schupbach T & Stein D (2000) Windbeutel is required for function and correct subcellular localization of the Drosophila patterning protein Pipe. Development 127, 5541–5550. 11076773 10.1242/dev.127.24.5541

[79] Barnewitz K , Guo C , Sevvana M , Ma Q , Sheldrick GM , Soling HD & Ferrari DM (2004) Mapping of a substrate binding site in the protein disulfide isomerase‐related chaperone wind based on protein function and crystal structure. J Biol Chem 279, 39829–39837. 15252019 10.1074/jbc.M406839200

[80] Lippert U , Diao D , Barak NN & Ferrari DM (2007) Conserved structural and functional properties of D‐domain containing redox‐active and ‐inactive protein disulfide isomerase‐related protein chaperones. J Biol Chem 282, 11213–11220. 17296603 10.1074/jbc.M604440200

[81] Monnat J , Neuhaus EM , Pop MS , Ferrari DM , Kramer B & Soldati T (2000) Identification of a novel saturable endoplasmic reticulum localization mechanism mediated by the C‐terminus of a Dictyostelium protein disulfide isomerase. Mol Biol Cell 11, 3469–3484. 11029049 10.1091/mbc.11.10.3469 PMC15007

[82] Pocanschi CL , Kozlov G , Brockmeier U , Brockmeier A , Williams DB & Gehring K (2011) Structural and functional relationships between the lectin and arm domains of calreticulin. J Biol Chem 286, 27266–27277. 21652723 10.1074/jbc.M111.258467 PMC3149320

[83] Brockmeier A , Brockmeier U & Williams DB (2009) Distinct contributions of the lectin and arm domains of calnexin to its molecular chaperone function. J Biol Chem 284, 3433–3444. 19074423 10.1074/jbc.M804866200

[84] Rizvi SM , Mancino L , Thammavongsa V , Cantley RL & Raghavan M (2004) A polypeptide binding conformation of calreticulin is induced by heat shock, calcium depletion, or by deletion of the C‐terminal acidic region. Mol Cell 15, 913–923. 15383281 10.1016/j.molcel.2004.09.001

[85] Sandhu N , Duus K , Jorgensen CS , Hansen PR , Bruun SW , Pedersen LO , Hojrup P & Houen G (2007) Peptide binding specificity of the chaperone calreticulin. Biochim Biophys Acta 1774, 701–713. 17499031 10.1016/j.bbapap.2007.03.019

[86] Brockmeier A & Williams DB (2006) Potent lectin‐independent chaperone function of calnexin under conditions prevalent within the lumen of the endoplasmic reticulum. Biochemistry 45, 12906–12916. 17042509 10.1021/bi0614378

[87] Saito Y , Ihara Y , Leach MR , Cohen‐Doyle MF & Williams DB (1999) Calreticulin functions in vitro as a molecular chaperone for both glycosylated and non‐glycosylated proteins. EMBO J 18, 6718–6729. 10581245 10.1093/emboj/18.23.6718 PMC1171734

[88] Jeffery E , Peters LR & Raghavan M (2011) The polypeptide binding conformation of calreticulin facilitates its cell‐surface expression under conditions of endoplasmic reticulum stress. J Biol Chem 286, 2402–2415. 21075854 10.1074/jbc.M110.180877 PMC3024734

[89] Lum R , Ahmad S , Hong SJ , Chapman DC , Kozlov G & Williams DB (2016) Contributions of the Lectin and polypeptide binding Sites of Calreticulin to its chaperone functions in vitro and in cells. J Biol Chem 291, 19631–19641. 27413183 10.1074/jbc.M116.746321 PMC5016696

[90] Horibe T , Gomi M , Iguchi D , Ito H , Kitamura Y , Masuoka T , Tsujimoto I , Kimura T & Kikuchi M (2004) Different contributions of the three CXXC motifs of human protein‐disulfide isomerase‐related protein to isomerase activity and oxidative refolding. J Biol Chem 279, 4604–4611. 14627699 10.1074/jbc.M310922200

[91] Jansen G , Maattanen P , Denisov AY , Scarffe L , Schade B , Balghi H , Dejgaard K , Chen LY , Muller WJ , Gehring K & *et al* (2012) An interaction map of endoplasmic reticulum chaperones and foldases. Mol Cell Proteomics 11, 710–723. 22665516 10.1074/mcp.M111.016550 PMC3434782

[92] Hayano T & Kikuchi M (1995) Molecular cloning of the cDNA encoding a novel protein disulfide isomerase‐related protein (PDIR). FEBS Lett 372, 210–214. 7556671 10.1016/0014-5793(95)00996-m

[93] Villani GR , Chierchia A , Di Napoli D & Di Natale P (2012) Unfolded protein response is not activated in the mucopolysaccharidoses but protein disulfide isomerase 5 is deregulated. J Inherit Metab Dis 35, 479–493. 22002444 10.1007/s10545-011-9403-8

[94] Vinaik R , Kozlov G & Gehring K (2013) Structure of the non‐catalytic domain of the protein disulfide isomerase‐related protein (PDIR) reveals function in protein binding. PLoS ONE 8, e62021. 23614004 10.1371/journal.pone.0062021 PMC3629029

[95] Baksh S , Burns K , Andrin C & Michalak M (1995) Interaction of calreticulin with protein disulfide isomerase. J Biol Chem 270, 31338–31344. 8537405 10.1074/jbc.270.52.31338

[96] Ushioda R , Hoseki J , Araki K , Jansen G , Thomas DY & Nagata K (2008) ERdj5 is required as a disulfide reductase for degradation of misfolded proteins in the ER. Science 321, 569–572. 18653895 10.1126/science.1159293

[97] Maattanen P , Gehring K , Bergeron JJ & Thomas DY (2010) Protein quality control in the ER: the recognition of misfolded proteins. Semin Cell Dev Biol 21, 500–511. 20347046 10.1016/j.semcdb.2010.03.006

[98] George G , Ninagawa S , Yagi H , Saito T , Ishikawa T , Sakuma T , Yamamoto T , Imami K , Ishihama Y , Kato K *et al* (2020) EDEM2 stably disulfide‐bonded to TXNDC11 catalyzes the first mannose trimming step in mammalian glycoprotein. ERAD. Elife 9. 10.7554/eLife.53455 PMC7039678 32065582

[99] Hirano M , Adachi Y , Ito Y & Totani K (2015) Calreticulin discriminates the proximal region at the N‐glycosylation site of Glc1Man9GlcNAc2 ligand. Biochem Biophys Res Commun 466, 350–355. 26362185 10.1016/j.bbrc.2015.09.026

[100] Blanco‐Herrera F , Moreno AA , Tapia R , Reyes F , Araya M , D'Alessio C , Parodi A & Orellana A (2015) The UDP‐glucose: glycoprotein glucosyltransferase (UGGT), a key enzyme in ER quality control, plays a significant role in plant growth as well as biotic and abiotic stress in Arabidopsis thaliana. BMC Plant Biol 15, 127. 26017403 10.1186/s12870-015-0525-2 PMC4465474

[101] Arnold SM & Kaufman RJ (2003) The noncatalytic portion of human UDP‐glucose: glycoprotein glucosyltransferase I confers UDP‐glucose binding and transferase function to the catalytic domain. J Biol Chem 278, 43320–43328. 12913004 10.1074/jbc.M305800200

[102] Takeda Y , Seko A , Hachisu M , Daikoku S , Izumi M , Koizumi A , Fujikawa K , Kajihara Y & Ito Y (2014) Both isoforms of human UDP‐glucose:glycoprotein glucosyltransferase are enzymatically active. Glycobiology 24, 344–350. 24415556 10.1093/glycob/cwt163

[103] Ito Y , Takeda Y , Seko A , Izumi M & Kajihara Y (2015) Functional analysis of endoplasmic reticulum glucosyltransferase (UGGT): Synthetic chemistry's initiative in glycobiology. Semin Cell Dev Biol 41, 90–98. 25481681 10.1016/j.semcdb.2014.11.011

[104] Tax G , Lia A , Santino A & Roversi P (2019) Modulation of ERQC and ERAD: a broad‐spectrum spanner in the works of cancer cells? J Oncol, 2019, 1–14. 10.1155/2019/8384913 PMC6791201 31662755

[105] Zhu T , Satoh T & Kato K (2014) Structural insight into substrate recognition by the endoplasmic reticulum folding‐sensor enzyme: crystal structure of third thioredoxin‐like domain of UDP‐glucose:glycoprotein glucosyltransferase. Sci Rep 4, 7322. 25471383 10.1038/srep07322 PMC4255179

[106] Persson K , Ly HD , Dieckelmann M , Wakarchuk WW , Withers SG & Strynadka NC (2001) Crystal structure of the retaining galactosyltransferase LgtC from Neisseria meningitidis in complex with donor and acceptor sugar analogs. Nat Struct Biol 8, 166–175. 11175908 10.1038/84168

[107] Ohara K , Takeda Y , Daikoku S , Hachisu M , Seko A & Ito Y (2015) Profiling aglycon‐recognizing sites of UDP‐glucose:glycoprotein glucosyltransferase by means of squarate‐mediated labeling. Biochemistry 54, 4909–4917. 26196150 10.1021/acs.biochem.5b00785

[108] Funkner A , Parthier C , Schutkowski M , Zerweck J , Lilie H , Gyrych N , Fischer G , Stubbs MT & Ferrari DM (2013) Peptide binding by catalytic domains of the protein disulfide isomerase‐related protein ERp46. J Mol Biol 425, 1340–1362. 23376096 10.1016/j.jmb.2013.01.029

[109] Labunskyy VM , Ferguson AD , Fomenko DE , Chelliah Y , Hatfield DL & Gladyshev VN (2005) A novel cysteine‐rich domain of Sep15 mediates the interaction with UDP‐glucose:glycoprotein glucosyltransferase. J Biol Chem 280, 37839–37845. 16129668 10.1074/jbc.M508685200

[110] Gladyshev VN , Jeang KT , Wootton JC & Hatfield DL (1998) A new human selenium‐containing protein. Purification, characterization, and cDNA sequence. J Biol Chem 273, 8910–8915. 9535873 10.1074/jbc.273.15.8910

[111] Korotkov KV , Kumaraswamy E , Zhou Y , Hatfield DL & Gladyshev VN (2001) Association between the 15‐kDa selenoprotein and UDP‐glucose:glycoprotein glucosyltransferase in the endoplasmic reticulum of mammalian cells. J Biol Chem 276, 15330–15336. 11278576 10.1074/jbc.M009861200

[112] Ferguson AD , Labunskyy VM , Fomenko DE , Arac D , Chelliah Y , Amezcua CA , Rizo J , Gladyshev VN & Deisenhofer J (2006) NMR structures of the selenoproteins Sep15 and SelM reveal redox activity of a new thioredoxin‐like family. J Biol Chem 281, 3536–3543. 16319061 10.1074/jbc.M511386200

[113] Lundstrom J & Holmgren A (1993) Determination of the reduction‐oxidation potential of the thioredoxin‐like domains of protein disulfide‐isomerase from the equilibrium with glutathione and thioredoxin. Biochemistry 32, 6649–6655. 8329391 10.1021/bi00077a018

[114] Mossner E , Huber‐Wunderlich M & Glockshuber R (1998) Characterization of *Escherichia coli* thioredoxin variants mimicking the active‐sites of other thiol/disulfide oxidoreductases. Protein Sci 7, 1233–1244. 9605329 10.1002/pro.5560070519 PMC2144011

[115] Yim SH , Everley RA , Schildberg FA , Lee SG , Orsi A , Barbati ZR , Karatepe K , Fomenko DE , Tsuji PA , Luo HR *et al* (2018) Role of Selenof as a Gatekeeper of Secreted Disulfide‐Rich Glycoproteins. Cell Rep 23, 1387–1398. 29719252 10.1016/j.celrep.2018.04.009 PMC9183203

[116] Ellgaard L , Bettendorff P , Braun D , Herrmann T , Fiorito F , Jelesarov I , Guntert P , Helenius A & Wuthrich K (2002) NMR structures of 36 and 73‐residue fragments of the calreticulin P‐domain. J Mol Biol 322, 773–784. 12270713 10.1016/s0022-2836(02)00812-4

[117] Moreau C , Cioci G , Iannello M , Laffly E , Chouquet A , Ferreira A , Thielens NM & Gaboriaud C (2016) Structures of parasite calreticulins provide insights into their flexibility and dual carbohydrate/peptide‐binding properties. IUCrJ 3, 408–419. 10.1107/S2052252516012847 PMC5094443 27840680

